# The Role of Emerging Risk Factors in Cardiovascular Outcomes

**DOI:** 10.1007/s11883-017-0661-2

**Published:** 2017-05-06

**Authors:** Ben Lacey, William G. Herrington, David Preiss, Sarah Lewington, Jane Armitage

**Affiliations:** 1 Clinical Trial Service Unit and Epidemiological Studies Unit (CTSU), Nuffield Department of Population Health, University of Oxford, Richard Doll Building, Old Road Campus, Roosevelt Drive, Oxford, OX3 7LF UK; 2 MRC Population Health Research Unit (MRC PHRU), Richard Doll Building, Old Road Campus, Roosevelt Drive, Oxford, OX3 7LF UK

**Keywords:** Epidemiology, Atherosclerosis, Vascular disease, Coronary heart disease, Stroke, Risk factors

## Abstract

**Purpose of Review:**

This review discusses the recent evidence for a selection of blood-based emerging risk factors, with particular reference to their relation with coronary heart disease and stroke.

**Recent Findings:**

For lipid-related emerging risk factors, recent findings indicate that increasing high-density lipoprotein cholesterol is unlikely to reduce cardiovascular risk, whereas reducing triglyceride-rich lipoproteins and lipoprotein(a) may be beneficial. For inflammatory and hemostatic biomarkers, genetic studies suggest that IL-6 (a pro-inflammatory cytokine) and several coagulation factors are causal for cardiovascular disease, but such studies do not support a causal role for C-reactive protein and fibrinogen. Patients with chronic kidney disease are at high cardiovascular risk with some of this risk not mediated by blood pressure. Randomized evidence (trials or Mendelian) suggests homocysteine and uric acid are unlikely to be key causal mediators of chronic kidney disease-associated risk and sufficiently large trials of interventions which modify mineral bone disease biomarkers are unavailable. Despite not being causally related to cardiovascular disease, there is some evidence that cardiac biomarkers (e.g. troponin) may usefully improve cardiovascular risk scores.

**Summary:**

Many blood-based factors are strongly associated with cardiovascular risk. Evidence is accumulating, mainly from genetic studies and clinical trials, on which of these associations are causal. Non-causal risk factors may still have value, however, when added to cardiovascular risk scores. Although much of the burden of vascular disease can be explained by ‘classic’ risk factors (e.g. smoking and blood pressure), studies of blood-based emerging factors have contributed importantly to our understanding of pathophysiological mechanisms of vascular disease, and new targets for potential therapies have been identified.

## Introduction

Major risk factors for cardiovascular disease include, but are not limited to, cigarette smoking, blood pressure, blood lipids, diabetes mellitus and adiposity [1–5, 6•]. The causality of these ‘classic’ risk factors is well established and they are commonly used to assess absolute cardiovascular risk in the general population [7–9]. Over the last few decades, however, other risk factors (commonly blood-based biomarkers) have been identified with potentially important implications for cardiovascular disease prevention, either through improved risk prediction or for treating cardiovascular disease (Table 1) [6•, 10]. For many of these risk factors, their causal relevance to cardiovascular disease is not well established and research is ongoing. This review will discuss the recent evidence for a selection of blood-based emerging risk factors, with particular reference to their relation with major cardiovascular outcomes, namely coronary heart disease and stroke.

**Table 1:** Emerging risk factors for atherosclerotic cardiovascular disease

Lipid-related biomarkers
High-density lipoprotein cholesterol
Triglycerides
Lipoprotein(a)
Apolipoprotein A1 and B
Lipoprotein-associated phospholipase A_2_
Inflammatory biomarkers
C-reactive protein
Interleukin (IL)-1, IL-6, IL-18
Tumour necrosis factor-α
Matrix metalloproteinase-9
Soluble CD40 ligand
Vascular and cellular adhesion molecule
Leukocyte count
Biomarkers of hemostasis and thrombosis
Fibrinogen
Coagulation factors II, V and VIII
von Willebrand factor antigen
Tissue plasminogen activator (t-PA)
Plasminogen activator inhibitor-1
D-dimer
Cardiac-related biomarkers
High sensitivity troponin
B-type natriuretic peptide
Kidney-related biomarkers
Creatinine
Microalbuminuria
Cystatin C
Calcium
Phosphate/fibroblast growth factor 23
Uric acid
Other factors
Homocysteine
Vitamin D
Metabolism-related (e.g. HbA1c)
Platelet-related factors (e.g. platelet volume)
Endothelial dysfunction (e.g. nitric oxide)
Environmental exposures (e.g. air pollution, radiation)
Other non-invasive measures of vascular disease (e.g. carotid plaque)

## Lipid-Related Biomarkers

Total cholesterol, low-density lipoprotein (LDL) cholesterol, high-density lipoprotein (HDL) cholesterol and non-HDL cholesterol (calculated as the difference between total cholesterol and HDL cholesterol) display robust log-linear associations with, and are considered classic predictors of, cardiovascular disease [4, 11]. All commonly used cardiovascular risk scores contain various combinations of these routinely measured lipids [8]. It has been demonstrated that the predictive capacities of apolipoprotein B100 and apolipoprotein A1 are very similar to non-HDL cholesterol and HDL cholesterol, respectively [4]. Robust data from monogenic conditions like familial hypercholesterolemia, randomized trials (most notably of statin therapy) and genetic studies have confirmed that LDL cholesterol is causally related to cardiovascular disease, and cardiovascular outcome trials of new powerful LDL cholesterol lowering therapies are starting to emerge [12–16]. Recent studies have also provided further insights into the relevance of HDL cholesterol, triglycerides, lipoprotein(a) and lipoprotein-associated phospholipase A2 to the development of cardiovascular disease.

### HDL Cholesterol, Apolipoprotein A1 and Triglycerides

The inverse associations of HDL cholesterol and apolipoprotein A1 with cardiovascular disease have led to the development of various therapeutic approaches to increase their levels [11, 17, 18]. While some early fibrate trials (medicines which reduce triglyceride, modestly increase HDL cholesterol and reduce LDL cholesterol) suggested cardiovascular benefit, recent larger studies of HDL cholesterol-raising therapies have yielded little or no benefit [19, 20•]. Cholesterol-ester transfer protein (CETP) inhibitors are able to increase HDL cholesterol by 30–120%, though it should be noted that potent CETP inhibitors also modestly reduce LDL cholesterol. Despite evidence from genetic studies indicating that those with genetically determined lower CETP activity may be at lower cardiovascular risk [21], three major outcomes trials of CETP inhibitors have shown no benefit [22, 23]. Results for the final ongoing major trial are expected in 2017. Of the three agents which failed, dalcetrapib (a weak inhibitor of CETP) has offered the purest test of the ‘HDL hypothesis’ given that it increases HDL cholesterol by 30% but has no effect on LDL cholesterol levels. In the DalOUTCOMES trial conducted in 15,871 participants following a recent acute coronary syndrome, dalcetrapib had no effect on cardiovascular events compared to placebo over 2.6 years [23]. Recent analyses of genetic variants have suggested that higher genetically determined HDL cholesterol is not associated with lower cardiovascular risk [14•, 24], implying that therapeutic interventions designed solely to increase HDL cholesterol are unlikely to provide cardiovascular benefit. It has also been announced that the development of apolipoprotein A1 Milano, an HDL mimetic given by weekly intravenous infusion, has been halted due to failure to reduce coronary atherosclerosis as measured by intravascular ultrasound [25].

**Fig. 1:**
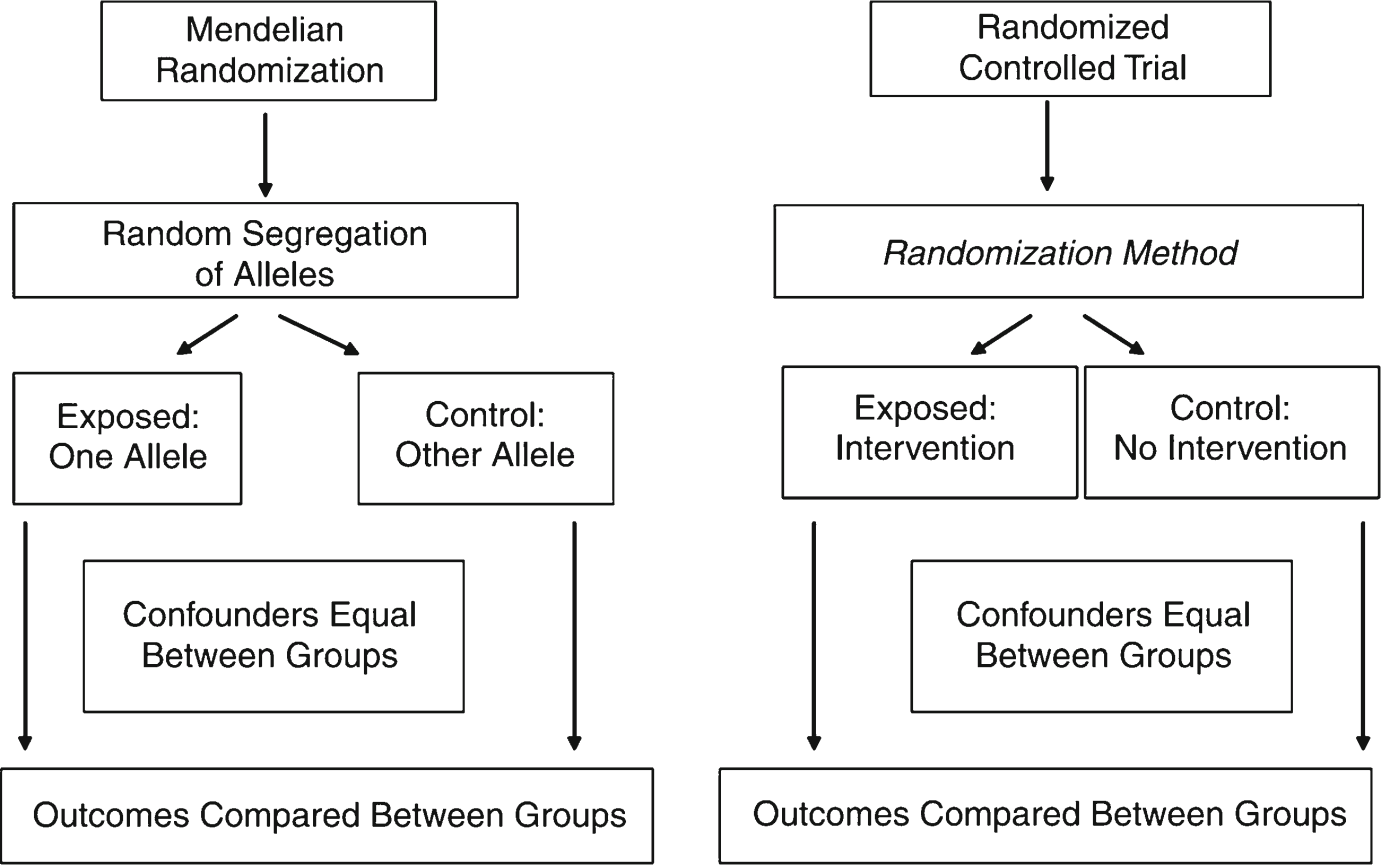
Mendelian randomization and randomized controlled trial designs compared. Reproduced with permission from: Davey Smith G, Ebrahim S. Mendelian Randomization: Genetic Variants as Instruments for Strengthening Causal Inference in Observational Studies. In: Biosocial Surveys, National Research Council of the National Academy of Sciences, 2008. Courtesy of National Academies Press, Washington, D.C

By contrast with HDL cholesterol, triglyceride levels are only weakly associated with, and do not improve prediction of, cardiovascular disease after adjustment for classic risk factors including HDL cholesterol [4]. However, recent Mendelian randomization studies (Fig. 1) have suggested that triglyceride-rich lipoproteins may be causally implicated in the development of cardiovascular disease. Lipoprotein lipase and apolipoprotein C3 are intimately involved in triglyceride metabolism. Genetic polymorphisms in both the *LPL* and *APOC3* genes which result in higher triglyceride concentrations have been demonstrated to be associated with increased risk of myocardial infarction, while approaches combining data from multiple variants that affect triglyceride levels have yielded similar results [14•, 26–28]. An injectable antisense oligonucleotide to apolipoprotein C3 is currently under investigation in clinical trials to determine its effect on triglycerides, though not yet on cardiovascular disease risk [29].

### Lipoprotein(a)

**Fig. 2:**
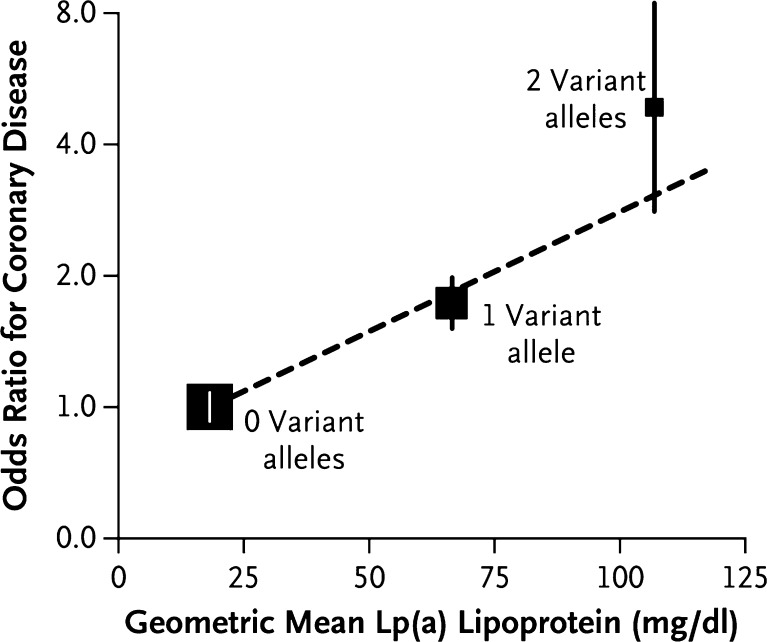
Association of the LPA Genotype Score with the Lp(a) Lipoprotein Level and the Risk of Coronary Disease in the PROCARDIS Cohort. The odds ratios (*squares*, with the size inversely proportional to the sampling variation) are for the association of the LPA genotype score (no variant alleles, one variant allele, or two variant alleles) with the risk of coronary disease, as measured with the use of ‘floating absolute risks’ which summarize the sampling variation for the three genotype scores without the selection of an arbitrary baseline genotype score. The vertical lines indicate 95% confidence intervals. Reproduced with permission from: Clarke R, Peden JF, Hopewell JC, et al. Genetic variants associated with Lp(a) lipoprotein level and coronary disease. N Engl J Med. 2009;361(26):2518–2528. Copyright © 2009 Massachusetts Medical Society. Reprinted with permission from Massachusetts Medical Society

Lipoprotein(a) (Lp[a]) is a lipoprotein similar in structure to LDL but with the addition of apolipoprotein(a) which is covalently bound to the apolipoprotein B of the lipoprotein. The apolipoprotein(a) varies in size depending on the number of kringle repeats that it contains. Lp(a) levels are highly heritable and are considerably higher in black individuals than in whites [30, 31]. Levels of Lp(a) are positively associated with coronary heart disease in a curvilinear fashion with a clear increase above 1 μmol/L [31], a relationship largely unaffected by adjustment for classic cardiovascular risk factors. Lp(a) appears to have a weaker association with stroke. It is not clear whether Lp(a) meaningfully improves the prediction of cardiovascular events. However, studies of genetic polymorphisms in the *LPA* gene indicate that Lp(a) is causally implicated in the development of cardiovascular disease (Fig. 2) [32]. An injectable antisense oligonucleotide to apolipoprotein(a) has been developed and shown to reduce circulating Lp(a) by around 70% in early phase clinical trials [33•].

### Lipoprotein-Associated Phospholipase A2

Lipoprotein-associated phospholipase A2 (Lp-PLA2) is a mediator expressed in atherosclerotic plaque which increases the production of pro-inflammatory and pro-apoptotic mediators. A case-control analysis of 1740 participants in the WOSCOPS trial cohort demonstrated that those in the highest quintile of Lp-PLA2 mass were at double the risk of a coronary event compared to those in the lowest quintile [34]. Subsequent meta-analyses confirmed that Lp-PLA2, whether measured as mass or activity, is modestly associated with risk of coronary events [35]. However, genetic and clinical trial evidence has established that inhibition of this pathway is unlikely to provide cardiovascular benefit [36, 37]. For example, darapladib, an inhibitor of Lp-PLA2, failed to reduce cardiovascular events in the STABILITY trial of 15,828 patients with stable coronary heart disease over 3.7 years [37].

## Inflammatory Biomarkers

Inflammatory processes play an important role in the pathogenesis of atherosclerotic vascular disease [38]. Yet, there is only limited evidence for the causal relevance of circulating inflammatory biomarkers on risk of vascular disease, and clinical trials of drugs specifically targeting inflammatory pathways are ongoing [39, 40].

### C-reactive Protein

C-reactive protein (CRP) is an acute-phase reactant, synthesized primarily in the liver and released into the blood in response to tissue injury or infection. A large meta-analysis of individual participant data reported that 1-standard deviation (SD) higher log_e_ CRP concentration was associated with 37% (95% CI 27–48%) higher risk of coronary heart disease and 27% (15–40%) higher risk of ischemic stroke, after adjusting for classic vascular risk factors [41]. However, a meta-analysis of Mendelian randomization studies found that genes encoding for CRP were not associated with risk of coronary heart disease (risk ratio for coronary heart disease 1.00 [95% CI 0.90–1.13] per 1-SD higher genetically raised log_e_ CRP) [42]. In light of these findings, CRP is unlikely to be causally related to vascular disease, but it may still have some utility in improving the predictive ability of cardiovascular risk scores (although the added value appears small) [43].

### Interleukin

Interleukin(IL)-6 is a pro-inflammatory cytokine that acts high in the inflammatory pathways (an ‘upstream’ inflammatory biomarker) with effects that include stimulation of hepatic acute-phase reactants, such as CRP [44]. It has been found to be strongly associated with coronary heart disease risk: a meta-analysis of 17 studies (5730 cases and 19,038 controls) reported an odds ratio for coronary heart disease, adjusting for several classic vascular risk and correcting for within-person variability, of 1.83 (95% CI 1.56–2.14) per 1-SD increase in usual IL-6 values [45]. Furthermore, meta-analyses of Mendelian randomization studies of an IL-6 receptor variant (Asp358Ala), with effects consistent to IL-6 receptor blockade, have reported a decreased risk of coronary heart disease per allele, supporting the causal role of the IL-6 pathway in coronary heart disease [46•, 47•].

### Other Pro-Inflammatory Cytokines

There is more limited evidence for the association of other pro-inflammatory cytokines with vascular disease. A recent large meta-analysis assessed the association between coronary heart disease risk and several other pro-inflammatory cytokines, including IL-18, matrix metalloproteinase-9, soluble CD40 ligand and tumour necrosis factor-α (TNF-α) [48]. Positive associations were described for IL-18 and TNF-α only, with relative risks of coronary heart disease per 1-SD higher levels of 1.13 (95% CI 1.05–1.20) and 1.17 (1.09–1.25), respectively. These associations have yet to be assessed reliably in Mendelian randomized studies. However, a Mendelian randomized analysis of the gene encoding a different cytokine, the IL-1 receptor antagonist, reported a per-allele odds ratio for coronary heart disease of 1.03 (95% CI 1.02–1.04) but no association with ischemic stroke (odds ratio 1.00 [0.98–1.02]) [49]. Trials are ongoing of drugs targeting the signalling pathways of IL-1, as well as those for IL-6 and TNF-α [39, 40, 50].

## Biomarkers of Hemostasis and Thrombosis

Several circulating biomarkers of hemostasis and thrombosis are strongly associated with risk of vascular disease. Mendelian randomization studies support a causal role for a number of factors involved with coagulation pathways, consistent with the efficacy of anticoagulant drugs in reducing atherosclerotic vascular events [51].

### Fibrinogen

The most extensively studied hemostatic biomarker is fibrinogen, the major circulating clotting factor by mass. A meta-analysis of prospective observational studies reported that, after adjusting for classic vascular risk factors, 1-g/L increase in usual plasma fibrinogen was associated with a 82% (95% CI 60–106%) higher risk of coronary heart disease and 82% (54–116%) higher risk of stroke [52]. Mendelian randomization studies, however, have not supported the causality of this association. A large meta-analysis of such studies reported a relative risk of coronary heart disease of 1.00 (95% CI 0.95–1.04) per higher-fibrinogen allele [51]. Inflammatory and hemostatic processes are interrelated and the strong observational associations of fibrinogen and vascular risk may, in part, reflect its regulation by pro-inflammatory cytokines, such as IL-6 [53]. As with CRP, however, fibrinogen may still be a useful adjunct to standard cardiovascular risk scores [43].

### Tissue Plasminogen Activator, D-dimer and von Willebrand Factor

Several prospective studies have described associations of other hemostatic factors and vascular disease. In particular, increased circulating levels of tissue plasminogen activator (t-PA), D-dimer and von Willebrand factor (VWF) have been associated with increased coronary heart disease risk [54–57]. A meta-analysis of prospective population-based studies found relative risks for coronary heart disease per 1-SD higher baseline levels of 1.13 (95% CI 1.06–1.21) with t-PA, 1.23 (1.16–1.32) with D-dimer and 1.16 (1.10–1.22) for VWF [58]. However, there was strong potential for residual confounding in this meta-analysis and, as such, the relation with coronary heart disease is still uncertain. The largest single study in this meta-analysis, which adjusted for a more comprehensive set of potential confounders, reported somewhat shallower associations (relative risks per 1-SD higher baseline levels of 1.07 [95% CI 0.99–1.14] with t-PA, 1.06 [1.00–1.13] with D-dimer and 1.08 [1.02–1.15] for VWF) [58].

### Other Hemostatic Factors

A genetic study of the effect of seven polymorphisms, all of which alter hemostatic pathways, reported strong associations with coronary heart disease risk for two genes both encoding for coagulant factors [59]. Per-allele relative risks for coronary heart disease of factor V (G1691A) and prothrombin (factor II; G20210A) were 1.17 (95% CI 1.08–1.28) and 1.31 (1.12–1.52), respectively. There was no evidence of an association with several platelet receptors (GP1a, GP1bα and GPIIIa) and the findings for genetic variants of plasminogen activator inhibitor-1 (PAI-1; a protein involved in fibrinolysis) were inconclusive as there was strong evidence of publication bias. There is limited evidence for the associations between other biomarkers of hemostasis and vascular disease [53].

## Cardiac-Related Biomarkers

Troponin and natriuretic peptides are released into the circulation from cardiac tissue even under physiological conditions, suggesting that they may be attractive cardiovascular biomarkers. The increasing availability of high-sensitivity assays for troponin in routine biochemistry laboratories (where it is typically used for the diagnosis of myocardial infarction) alongside brain natriuretic peptide (especially N-terminal pro-b-type natriuretic peptide [NT-proBNP] which is usually measured when left ventricular failure is suspected), indicates that these assays could be easily incorporated into routine cardiovascular risk screening if they are demonstrated to predict risk effectively.

The Natriuretic Peptides Studies Collaboration combined data from 40 studies for 95,000 participants without cardiovascular disease at baseline. Those with NT-proBNP levels in the highest tertile were at double the risk of suffering a coronary event, stroke, or developing heart failure [60•]. Addition of NT-proBNP to a risk model with classical risk factors yielded modest improvements in cardiovascular risk prediction, similar in scale to that provided by HDL cholesterol and superior to CRP. Neprilysin inhibitors (e.g. sacubitril) are agents which inhibit the degradation of natriuretic peptides and other endogenous vasoactive peptides. The PARADIGM-HF trial, conducted in 8442 patients with heart failure, confirmed that neprilysin inhibition (when added to renin-angiotensin system blockade) reduces hospitalization and death due to heart failure but probably has little effect on the risk of stroke or myocardial infarction [61].

Data regarding the predictive capacity of high sensitivity troponin (both troponin-T and troponin-I) in those without cardiovascular disease are more limited than for NT-proBNP. In one major study, troponin-T levels were related to outcomes in 9698 participants without cardiovascular disease aged 54 to 74 years [62]. One third had measurable troponin-T. There was a graded increase in cardiovascular events at progressively higher levels of troponin-T compared to those with unmeasurable levels and the authors concluded that its predictive capacity was similar to that of NT-proBNP.

## Chronic Kidney Disease and Kidney-Related Factors

Chronic kidney disease (CKD) is defined and staged by severity of reduced renal function, usually quantified using estimated glomerular filtration rate (eGFR) [63]. Its prevalence reaches 10% in populations where old age or diabetes are common [64]. CKD is independently associated with substantially increased risk of cardiovascular disease, with progressively more advanced CKD associated with progressively higher risk [65, 66].

The spectrum of cardiovascular disease which manifests in people with CKD is wide and includes both arterial and cardiac disease. Common presentations of arterial disease in those with CKD include intimal atherosclerotic lesions [67], non-atheromatous non-calcified arterial stiffening [68], and heavy medial calcification [69] (see Wheeler et al. [70] for a comprehensive review). Correspondingly, people with CKD are at increased risk of coronary artery disease and structural heart disease [66, 71, 72]. CKD is also associated with increased stroke risk, and for large vessel stroke, there is some Mendelian randomization evidence that this association may be one of cause and effect. [73].

Every 30% decrement in eGFR is associated with about a 30% increase in risk of a cardiovascular event, so that a reduction in eGFR from 60 to 10 mL/min/1.73m^2^ is associated with about fourfold increased risk of cardiovascular disease [65]. The kidneys have a key role in modulating blood pressure which is a clear mechanism by which CKD causes increased risk of cardiovascular disease [74]. Each 10 mL/min/1.73m^2^ lower eGFR is associated with about a 5 mmHg higher systolic blood pressure [75], so a reduction in eGFR from 60 to 10 mL/min/1.73m^2^ would be expected to increase SBP by at least 20 mmHg, approximately doubling cardiovascular risk [2, 74]. The effect of CKD on blood pressure might therefore account for up to about one half of the association between CKD and cardiovascular disease.

A range of emerging risk factors which correlate with reduced renal function have been proposed to mediate the remaining CKD-associated cardiovascular risk which is not explained by blood pressure. However, the precise roles are not yet fully elucidated and quantified. For some, including homocysteine and uric acid, a body of evidence now suggests they are unlikely to be key causal mediators of arterial disease. Other risk factors remain potential candidates, including mediators of accelerated arterial calcification and certain lipid abnormalities. Details of the evidence supporting each of these mechanisms are provided below, with an emphasis on effects on coronary artery disease.

Homocysteinuria is a rare inherited disorder of metabolism which causes high blood homocysteine concentration and premature cardiovascular disease. Reduced renal function leads to moderately increased blood homocysteine concentrations and it is estimated that a 5 μmol/L increase in homocysteine is observed for each 10 mL/min/1.73m^2^ lower eGFR. Observational studies suggest such a change might increase coronary risk by about 20% [76]. However, Mendelian randomization experiments have been negative, and a meta-analysis of trials testing homocysteine lowering using folate supplementation found no evidence of benefit on major cardiovascular events [77, 78]. Moderate elevations of homocysteine therefore seem unlikely to be causally associated with coronary artery disease.

Blood uric acid concentration also increase as renal function falls, and positive associations between uric acid levels and coronary artery disease have been observed [79, 80]. Each 10 mL/min/1.73m^2^ lower eGFR is associated with about a 10–15 μmol/L increase in uric acid concentration, which is predicted to increase coronary risk by about 10% [79, 80]. However, again, Mendelian randomization experiments have cast doubt about whether such associations are causal, as once pleotropic pathways were taken into account, no clear association was observed [81].

Heavy arterial calcification is a particular feature of advanced CKD [69]. Both intimal and medial calcifications are common and associated with increased risk of cardiovascular disease, with perhaps the volume of intimal atheromatous coronary plaque calcification being more important than the density of calcification [69, 82]. A key emerging risk factor for arterial calcification is blood phosphate, the concentration of which increases as the capacity for its urinary excretion falls. High blood phosphate concentration can directly induce ossification of vascular smooth muscle cells and is associated with increased arterial calcification [83, 84]. For each 0.3 mmol/L increase in phosphate (which is the approximate effect of each 10 mL/min/1.73m^2^ lower eGFR), there is about a 30% increased risk of cardiovascular disease [85]. Phosphate lowering is achieved by dietary modification and phosphate binding medication [70, 86]. However, sufficiently large placebo-controlled trial have not been performed to confirm cardiovascular benefit from this practice. Trials comparing different binders are also complicated to interpret, as there are possible adverse cardiovascular effects of calcium-containing binders, and beneficial cardiovascular effects mediated through lipid lowering with some of the non-calcium containing binders [87]. Nevertheless, lowering phosphate is deeply embedded in nephrology practice.

Fibroblast growth factor-23 (FGF23) has recently emerged as another potential mediator of cardiovascular risk in CKD. It has particularly drawn interest as associations are consistently strong and FGF23 concentrations rise early in CKD mirroring the early rise in cardiovascular risk which is apparent with only modest reductions in renal function [88, 89]. FGF23 serves to increase phosphate excretion and does not appear to cause arterial calcification, observations which both suggest FGF23 is a protective homeostatic hormone (like natriuretic peptides) [90]. However, there is some mechanistic evidence that FGF23 may be directly cardiotoxic [91, 92]. Randomized experiments are therefore required to confirm whether consistently positive observational associations between FGF23 and risk of coronary artery disease represent confounding, or are causal.

Statin-based therapy is effective at lowering atherosclerotic risk in CKD, [93, 94] but LDL cholesterol (with the exception of nephrotic syndrome) is not generally raised in CKD [86, 87]. Instead, each 10 mL/min/1.73m^2^ lower eGFR is associated with a modest reduction in HDL cholesterol, perhaps as much as 0.1 mmol/L, which is associated with a 5–10% increased risk of coronary artery disease [4]. As discussed above, current evidence does not support HDL cholesterol being causally related to coronary disease. Perhaps more promisingly, reduced renal function is also associated with increased concentration of Lp(a) particles [95]. Mendelian randomization experiments predict Lp(a) to be causally associated with coronary artery disease, particularly the small Lp(a) isoforms [32]. Each 10 mL/min/1.73m^2^ lower eGFR is associated with a 0.2–0.3 μmol/L increase in Lp(a), which might increase coronary artery disease risk by about 10% [95]. Some uncertainty remains as the raised Lp(a) in CKD is mainly of the large isoform type, whose relevance to coronary artery disease risk is less well understood, but emerging Mendelian randomization evidence suggests that Lp(a) concentration may be associated with coronary risk independent of Lp(a) particle size [96].

## Future Directions for Randomized Trials

Early stage clinical trials of drugs which lower lipoprotein(a) and triglycerides are in process, and the results to these studies will be influential in determining future trials of lipid-related factors [29, 33•]. In addition, novel mechanisms of reducing LDL cholesterol are being tested, including trials of PCSK9 inhibitors (monoclonal antibodies which target circulating PCSK9) [15, 16]. These antibodies have been found to dramatically lower LDL levels and, according to recently published results from the FOURIER trial, to reduce cardiovascular events in patients with clinically evident vascular disease [97]. With respect to inflammatory factors, there are a number of trials evaluating the effect of anti-inflammatory agents on cardiovascular outcomes, including low-dose methotrexate (a generic drug used to treat autoimmune conditions such as rheumatoid arthritis) and canakinumab (a human monoclonal antibody targeting the pro-inflammatory cytokine IL-1β) [39, 40, 50]. There is also a need for future trials of anti-inflammatory agents targeting specific cytokines for which there is strong evidence of causality, such as IL-6.

## Limitations of Review

This review focuses on selected blood-based biomarkers and their relation to major vascular disease. There are other important blood-based biomarkers that it was not possible to cover in this review, some of which are listed in Table 1. In addition, we did not address metabolomic approaches to the simultaneous measurement of hundreds to thousands of small molecules that may yet lead to the identification of novel biomarkers. Furthermore, this review focuses on associations with coronary heart disease and stroke and did not address the relation of emerging risk factors with other major manifestations of vascular disease, notably abdominal aortic aneurysm and peripheral vascular disease. It was also outside the scope of this review to discuss non-blood-based emerging risk factors for vascular disease, such as radiation, coronary artery calcification, carotid intima-media thickness, carotid plaque and ankle-brachial index [10]. For a recent wide-ranging review of emerging risk factors for stroke see Hopewell and Clarke [6•].

## Conclusions

This review has discussed a range of blood-based cardiovascular risk factors that have emerged over the last few decades, with a particular focus on their relation with major vascular disease. For some risk factors, there is now strong evidence that their association with coronary heart disease and stroke is not causal. Non-causal risk factors may still have value, however, when added to cardiovascular risk scores and a number of cardiac-related biomarkers hold particular promise in this regard. For other risk factors (including triglyceride-rich lipoproteins, lipoprotein(a), IL-6 and several coagulation factors), there is increasing evidence of a causal role in the pathogenesis of major vascular disease. Although much of the burden of vascular disease can be explained by classic risk factors, studies of emerging risk factors have contributed importantly to our understanding of the pathophysiological mechanisms of vascular disease, and new targets for potential therapies (for both primary and secondary prevention) have been identified.

## Abbreviations

APOC3: Apolipoprotein C3 gene
CETP: Cholesterol-ester transfer protein
CI: Confidence interval
CKD: Chronic kidney disease
CRP: C-reactive protein
eGFR: Estimated glomerular filtration rate
FGF23: Fibroblast growth factor-23
HDL: High-density lipoprotein
IL: Interleukin
Lp(a)/LPA: Lipoprotein(a)/Lp(a) gene
Lp-PLA2: Lipoprotein-associated phospholipase A2
LDL: Low-density lipoprotein
LPL: lipoprotein lipase gene
NT-proBNP: N-terminal pro-b-type natriuretic peptide
SD: Standard deviation
t-PA: Tissue plasminogen activator
TNF-α: tumour necrosis factor-α
VWF: von Willebrand factor
